# Correspondence

**Published:** 1907-09

**Authors:** 


### CORRESPONDENCE.

Vienna, Austria, August 1, 1907.

Editor Journal-Record of Medicine.

Dear Sir: The abundant and always available clinical material found in the hospitals of Europe offer strong inducements to the American physician to visit the principal medical and surgical centers of this quarter of the globe, for here rich rewards indeed gladly await his coming.

The poverty, the crime, the crowded, filthy and thoroughly unsanitary condition of some of the surburban and some of the manufacturing districts of the many great cities of the old world provide a perennial harvest of disease and injury which fills the vacated bed of the dead or convalescent hospital patient with scarce an interval in the endless chain of occupants.

The great Salpetriere, the Beaujou and the Broca, of Paris, afford an adequate idea of the representative French hospital. The buildings are, for the most part, old and dirty, the attendants are untidy and slovenly, the operative technique is careless and indifferent, and the general tone and atmosphere about these places are below par.

In Vienna, the visitor finds ready access to most excellent institutions. Here, the Algemeines Kraukenhaus, or general hospital, with its inexhaustible clinics—treating over two thousand patients daily—freely opens every department to the visitor, and the physician, surgeon or specialist meets in one building every conceivable ailment in unequaled profusion.

The splendid Royal Infirmary of Edinborough, with nine hundred beds, the thoroughly modern St. Thomas, of London, and also the older but superb model hospitals of St. Bartholomew and Guy, each treating an average of six hundred "in-patients" daily, are equipped in the most approved manner, and, being conducted in connection with medical schools of international reputation, present conditions in full accord with the most advanced standards of hospital management.

I have, within the last few weeks, enjoyed the opportunity of visiting all of these magnificent institutions and of witnessing the methods which obtain in each of them. It has been a source of great interest to study the details of management in their various departments, to see operations by distinguished representatives of the profession and to note the treatment of cases in the public wards; about all of which I shall have something to say at another time.

One is most forcibly impressed—almost bewildered—by the enormous amount of work done, day after day, in all of these large hospitals, particularly in the mammoth Vienna general hospital with its three-quarters of a million patients annually. But, at the same time, he cannot fail to recognize the superiority of the English and Scotch hospitals as compared with the German and especially as compared with the French, in cleanliness, order, general technique, and, not least, for the kindness, consideration and feeling manifested toward the patient on the part of the physicians and by all of the hospital attendants. This is in striking contrast to the treatment accorded patients in the other countries, and it is a distinctive feature to be most warmly commended. It seems to me, after careful observation, that the British hospitals are, in every respect, decidedly superior to the continental hospitals. They are certainly far ahead of the Parisian institutions in every essential particular.

From Vienna I shall go to Berlin, which you know is the finest city and one of the greatest medical centers in the world, and, while exact information for purposes of comparison is difficult to obtain, I expect to gather some facts which may be of general interest to the readers of the Journal.

Yours truly,

JAMES B. BAIRD, JR.

Editor Medical Journal;—

Allow me to express my particular agreement to the supposition, which you take over from the American Journal of Clinical Medicine, that there is such a disease as "Misokainia." But I must take exception to the opinion that sclerosis and congelation of the cerebral tissues and the heart are the "effects" of the disease. I am of the opinion that they are the cause of the disease, or one of the main causes.

Furthermore I believe, that the condition is to a great extent physiological. The tendency to combat new ideas and discoveries is an observation so exceedingly common, that we can not restrict it to pathology alone; it is like obstetric cases a physiological occurrence.

I am also inclined to believe that the condition is more one of the blood than one of the brain, and in the blood more one of the red corpuscles than of the heart. It is a condition, pathological and physiologial, which is not incompatible with fair functionating of the heart as well as the brain, otherwise. It is a disease of the will. It is a matter of idiosyncracy, greatly. Observation teaches, that there is a selection; the one has a predilection one way, the other another. It is most likely, that the red blood-corpuscles have something to do with it, these curious microscopical disks of which we know so little and whose bizarre histology suggests so unexpected actuality, explaining psycological points otherwise entirely enigmatical.

What I doubt, is, that the interest and the sympathy of the profession, in misokainia, can be awakened by misokainia: the very circumstance that misokainia is so widely spread, indicates, that the selection of such a hard term, as misokainia, is not the road to the least resistance.

Dr. C. A. F. Lindorme.

---

It is reported that the King of Spain has tuberculosis, and the Spanish Court is very uneasy regarding his condition.

